# Organic Anisotropic Excitonic Optical Nanoantennas

**DOI:** 10.1002/advs.202201907

**Published:** 2022-05-26

**Authors:** Evan S. H. Kang, Sriram KK, Inho Jeon, Jehan Kim, Shangzhi Chen, Kyoung‐Ho Kim, Ka‐Hyun Kim, Hyun Seok Lee, Fredrik Westerlund, Magnus P. Jonsson

**Affiliations:** ^1^ Department of Physics Chungbuk National University Cheongju 28644 Republic of Korea; ^2^ Laboratory of Organic Electronics Department of Science and Technology (ITN) Linköping University Norrköping 60174 Sweden; ^3^ Department of Biology and Biological Engineering Chalmers University of Technology Gothenburg 41296 Sweden; ^4^ Pohang Accelerator Laboratory Pohang University of Science and Technology Pohang 37673 Republic of Korea

**Keywords:** hyperbolic polaritons, J‐aggregates, localized surface exciton resonances, Mie resonances, nanoantennas

## Abstract

Optical nanoantennas provide control of light at the nanoscale, which makes them important for diverse areas ranging from photocatalysis and flat metaoptics to sensors and biomolecular tweezing. They have traditionally been limited to metallic and dielectric nanostructures that sustain plasmonic and Mie resonances, respectively. More recently, nanostructures of organic J‐aggregate excitonic materials have been proposed capable of also supporting nanooptical resonances, although their advance has been hampered from difficulty in nanostructuring. Here, the authors present the realization of organic J‐aggregate excitonic nanostructures, using nanocylinder arrays as model system. Extinction spectra show that they can sustain both plasmon‐like resonances and dielectric resonances, owing to the material providing negative and large positive permittivity regions at the different sides of its exciton resonance. Furthermore, it is found that the material is highly anisotropic, leading to hyperbolic and elliptic permittivity regions. Nearfield analysis using optical simulation reveals that the nanostructures therefore support hyperbolic localized surface exciton resonances and elliptic Mie resonances, neither of which has been previously demonstrated for this type of material. The anisotropic nanostructures form a new type of optical nanoantennas, which combined with the presented fabrication process opens up for applications such as fully organic excitonic metasurfaces.

## Graphical abstract

Optical nanoantennas based on organic anisotropic J‐aggregate materials with strong exciton absorption provide dual functionality in the same nanostructure. They can support hyperbolic localized surface‐exciton polariton resonances on one spectral side of the exciton resonance while they support elliptic Mie resonances on the other side.

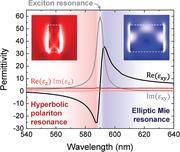

## 1 Introduction

Nanostructured materials are essential building blocks in nanooptics. Their unique ability to manipulate light at the nanoscale, which cannot be found in their non‐structured counterparts, facilitates a multitude of applications including biosensing, flat optics, holography, subwavelength super‐resolution imaging, etc.^[^ ^1^, ^2^, ^3^, ^4^, ^5^ ^]^ Two types of optical nanostructures, of completely different origin, have been extensively studied. The first type is metallic nanostructures, strongly interacting with light via excitation of their localized surface plasmon resonance (LSPR).^[^ ^6^, ^7^ ^]^ The origin of the plasmonic response is the negative real part of the permittivity of metallic materials, where the restoring forces on free charge carriers lead to resonant collective oscillations. The resulting electric field enhancement is confined in the surrounding area outside the nanostructures. While plasmonics research has primarily been restricted to inorganic metals like gold and silver, we recently introduced organic conducing polymers for a new type of dynamic plasmonics based on redox tuning.^[^ ^8^ ^]^ The other main type of nanooptics is based on dielectric nanostructures made of high refractive index materials.^[^ ^9^, ^10^, ^11^ ^]^ They can also strongly interact with light, but via Mie resonances instead of plasmons. The origin of Mie resonances is the optical geometry of the nanostructures. For example, the magnetic dipole resonance, which is the lowest order Mie resonance, occurs when the wavelength of light confined inside the nanostructures becomes comparable to their optical dimension ($\lambda \approx \sqrt{\varepsilon }d$, where *ε* is the relative permittivity of the non‐magnetic material and *d* is the size of the nanostructure).^[^ ^10^ ^]^ Materials with high refractive index, and thus large positive permittivity, are required for nanostructures to support Mie resonances in the visible spectral range.^[^ ^4^, ^5^ ^]^ For high resonance quality factors it is also essential to have a large contrast in refractive index between the nanostructure and the surrounding media, which lowers radiation leakage and improves field confinement.^[^ ^11^ ^]^

Organic excitonic materials with strong and narrow absorption, such as J‐aggregated molecules, have recently been proposed as alternatives to achieve metal‐like negative permittivity in certain spectral ranges, even though they do not have free charge carriers.^[^ ^12^ ^]^ The relative permittivity of such materials with a single Lorentz type exciton resonance can be described as

(1) $$\varepsilon \left(\omega \right)=\varepsilon_{b}+\frac{f\omega_{0}^{2}}{\omega_{0}^{2}-\omega^{2}-i\omega \gamma }$$

where *ω*_0_ is the resonant angular frequency, *f* is the reduced oscillator strength of the excitonic transition, *γ* is the damping rate and *ε*_*b*_ is the nonresonant background permittivity of the material. The imaginary part of the permittivity forms a peak responsible for the dissipative absorption of the material, whereas the real part of the permittivity shows a peak‐dip feature in consequence of the Kramers–Kronig relations. Interestingly, if the oscillator strength is large and the damping rate is small, the dip in the real permittivity can reach below zero, meaning that the material behaves optically like a metal in this region. This opens up for excitation of surface exciton polariton (SEP) resonances that resemble plasmons yet originate from electron‐hole pairs instead of free charge carriers as for plasmonic materials.^[^ ^12^ ^]^ Low temperature measurements were required for the observation of SEPs in inorganic materials, because of the binding energy of Wannier–Mott excitons being lower than room temperature thermal energy. By contrast, the high binding energy of Frenkel excitons in organic materials has enabled observation of propagating SEPs even at room temperature.^[^ ^12^, ^13^, ^14^, ^15^ ^]^

Nanostructured organic excitonic materials are of particular interest because they are expected to support localized surface exciton polaritons (LSEPs) with subwavelength light confinement, resembling LSPRs of metallic nanostructures. A series of optical calculation studies have predicted LSEPs in various organic excitonic nanostructures, including nanospheres,^[^ ^12^, ^16^ ^]^ nanodisks and nanodisk dimers,^[^ ^17^ ^]^ and core‐shell structures.^[^ ^18^ ^]^ Even surface lattice resonances in periodic arrays have been suggested, based on the assumption of existence of LSEPs.^[^ ^19^ ^]^ However, the realization of these systems and the associated observation of LSEPs have been hampered not least because of fabrication challenges. For instance, conventional photo‐/electron‐beam‐lithography techniques cannot be directly applied to these relatively vulnerable organic solids. Up to date, the most successful attempts at nanostructuring excitonic material is local bleaching of thin films via electron beam^[^ ^20^ ^]^ or femtosecond laser exposure.^[^ ^21^ ^]^

In this study, we experimentally realize well‐defined nanostructured J‐aggregate materials using solvent‐assisted nanoscale embossing (SANE)^[^ ^22^ ^]^ assisted by reactive ion etching (RIE).^[^ ^23^ ^]^ Atomic force microscopy (AFM) images confirm that this method is suitable for nanostructuring sensitive organic materials with sub‐hundred‐nm lateral resolution. The resulting nanostructures support not only plasmon‐like resonances, but also Mie‐like resonances, providing dual functionality. This intriguing result is attributed to the excitonic material having both a negative permittivity region where it behaves optically as a metal and a closely spaced spectral region with large positive permittivity where the material instead behaves as a high index dielectric. Furthermore, we show that the permittivity of the organic excitonic thin films is highly anisotropic, as attributed to an anisotropic nature of the J‐aggregate structures. As a result, the material provides hyperbolic (elliptic) isofrequency surface in the negative (positive) permittivity regions, respectively. The nanostructures thereby support hyperbolic LSEPs and elliptic Mie resonances, with spectra showing good agreement with simulated results using the finite‐difference time‐domain (FDTD) method. The simulations further reveal the characteristic nearfield distribution of the resonance modes in the anisotropic excitonic nanostructures.

## 2 Results and Discussion

**Figure 1:**
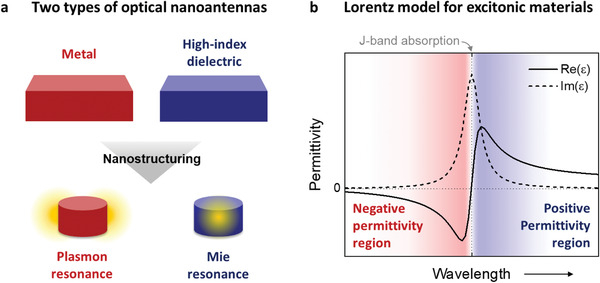
a) Schematic illustration of two basic types of optical nanoantennas: metallic nanoantennas that support localized surface plasmon resonances (left) and high‐index dielectric nanoantennas that support Mie resonances (right). The yellow areas indicate exemplified nearfield enhancements for plasmon and Mie resonances, with electric fields primarily being confined outside and inside the nanostructure, respectively. b) Example of complex permittivity of an excitonic material described by a single Lorentzian, showing an optically metallic negative permittivity region at wavelength shorter than the exciton resonance (red shaded area) and a large positive permittivity region at the other side of the exciton resonance (blue shaded area).

**Figure** **1** illustrates the main concept of dual resonance functionality in excitonic nanostructures. J‐aggregated materials exhibit a very narrow and intense absorption peak known as J‐band, redshifted from the monomer absorption, due to J‐aggregation. This gives the excitonic materials the interesting feature of having both a metal‐like negative permittivity region and a high‐index positive permittivity region, on each side of the J‐band (Figure 1b). We propose that nanostructuring such materials therefore can allow excitation of both plasmon‐like LSEP resonances and Mie resonances, even in the very same nanostructure, depending on dimensions.

As organic J‐aggregate excitonic material, our study employed the J‐aggregated cyanine dye TDBC (5,6‐dichloro‐2‐[[5,6‐dichloro‐1‐ethyl‐3‐(4‐sulfobutyl)benzimidazol‐2‐ylidene]propenyl]‐1‐ethyl‐3‐(4‐sulfobutyl)benzimidazolium hydroxide, inner salt, sodium salt). TDBC doped polyvinyl alcohol (PVA) films have been widely utilized especially when strong oscillator strength is necessary, such as in strong light‐matter coupling, because PVA increases the wettability and stability of TDBC, while maintaining the J‐aggregate structure of the TDBC. However, increasing PVA concentration inevitably leads to a decrease in the number of TDBC molecules per volume, which weakens the intensity of the J‐band absorption. To maintain maximum J‐band absorption, we therefore used pure TDBC throughout this study.

**Figure 2:**
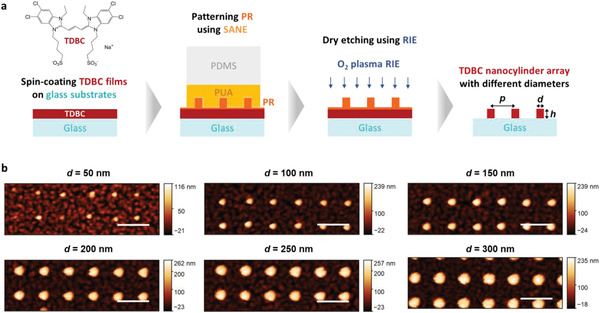
a) Fabrication scheme of TDBC nanocylinder arrays using SANE assisted with RIE, with the molecular structure of TDBC shown above the leftmost structure. The rightmost structure indicates the three characteristic dimensions of the nanocylinder arrays: periodicity *p*, diameter *d*, and height *h*. b) AFM images of resulting TDBC nanocylinder arrays with varying diameter and fixed periodicity at 800 nm. The scale bars are all 1 µm.

**Figure** **2**a schematically depicts the protocol for fabricating the TDBC nanostructures, starting with spin‐coating of 200–300 nm thick TDBC films on glass substrates. Next, we built a photoresist (PR) nanopattern on top of the TDBC film using SANE, employing a polyurethane acrylate (PUA) stamp containing arrays of nanoholes. The nanoholes all had a periodicity of 800 nm and a depth of 200 nm, but varying diameters from 50 to 300 nm (see Figure S1, Supporting Information for the fabrication process of the PUA stamps). The resulting PR nanopattern was then used as a mask for O_2_ plasma reactive ion etching (RIE) to form cylindrical TDBC nanostructure arrays with different diameters. AFM imaging after the final fabrication step confirmed that our method was able to successfully produce TDBC nanocylinders of the desired dimensions down to 50 nm in diameter (see Figure 2b and also Figure S2, Supporting Information for the complete set of AFM images and height profiles for both PR nanopattern on TDBC thin films and TDBC nanocylinders). The height of the nanocylinders was ≈200 nm for all diameters, except somewhat lower for the smallest diameter of 50 nm as attributed to incomplete formation of the PR nanopattern, which sets the resolution limit of our method. The very thin (<20 nm thick) background pattern between the nanocylinders are TDBC leftovers that remained after the etching. Extinction measurements outside of the arrays indicated that their contribution to the optical response was negligible (not shown), but we cannot completely exclude possible weak contribution as discussed more below. The overall structure of the fabricated TDBC nanocylinders was also confirmed by SEM and optical microscopy (Figures S3 and S4, Supporting Information).

**Figure 3:**
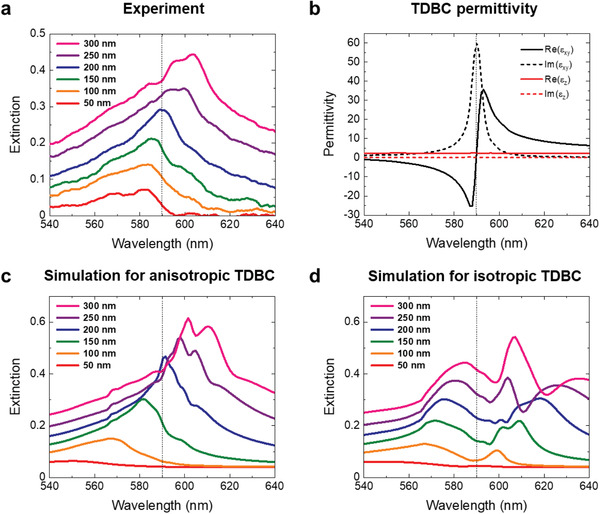
a) Experimentally measured extinction spectra for TDBC nanocylinder arrays with varying diameters. b) Anisotropic permittivity of TDBC obtained by ellipsometry measurements. Calculated extinction spectra using FDTD simulations assuming c) anisotropic and d) isotropic permittivity. Cylinder height of 150 nm and periodicity of 800 nm were used for the simulations. The vertical dashed lines indicate the exciton resonance position.

We acquired extinction spectra of TDBC nanocylinder arrays using a microscope coupled to a spectrometer via an optical fiber acting as a pinhole in the image plane. The incident light was linearly polarized along the array axis (results for unpolarized illumination showed highly similar results, Figure S5, Supporting Information). As shown in **Figure** **3**a, the TDBC nanocylinders exhibited extinction peaks that were not located at the exciton resonance (at ≈590 nm), but on either side of the exciton resonance. For diameters of <150 nm, the peaks were positioned in the negative permittivity region (on the left side of the exciton resonance in Figure 3a), which correspond to LSEP resonances. For larger diameters, on the other hand, the peaks appeared in the spectral region of large positive permittivity (on the right side of the exciton resonance), which corresponds to Mie resonances. We performed FDTD simulations to confirm and better understand the experimental results. To resemble the real system, we first carefully measured the permittivity of nonstructured thin TDBC films using ellipsometry, for three different thicknesses and five different angles (see Figure S6, Supporting Information for the ellipsometric raw data). By contrast to previously reported TDBC systems,^[^ ^12^, ^16^, ^17^, ^18^, ^19^ ^]^ the results revealed that the material permittivity of our TDBC films is strongly anisotropic (Figure 3b). While the in‐plane permittivity components (*ε*_xy_) are in accordance with a typical Lorentzian type exciton resonance (Equation (1)), the out‐of‐plane permittivity (*ε*_z_) showed almost no resonant features and positive values in the whole measured spectral range (see Figure S7, Supporting Information for wider spectral range and enlarged out‐of‐plane permittivity). This means that our TDBC films are naturally hyperbolic in the (in‐plane) negative permittivity region and elliptic in the large positive permittivity region. This striking feature has been mostly overlooked or only qualitatively addressed in previous studies exploiting TDBC,^[^ ^24^ ^]^ although it has been widely known that J‐aggregated TDBC molecules have highly ordered, directionally self‐assembled structures.^[^ ^25^, ^26^, ^27^ ^]^ Simulated nanocylinder extinction spectra using the measured anisotropic permittivity show good agreement with the experimental spectra (Figure 3c), which also confirms the anisotropic nature of the TDBC material. For comparison, we present simulated results assuming fictitious isotropic permittivity (setting *ε*_z_ = *ε*_xy_), which show distinct differences from the experiments (Figure 3d). It should be noted that the simulations in Figure 3 used 150 nm thick nanocylinders instead of 200 nm, which showed best agreement with the experimental results, but 200 nm thick nanocylinders showed qualitatively the same results (see Figure S8, Supporting Information). This may be due to the top part of the nanocylinders becoming bleached during fabrication. As a result, the effective height of the unbleached TDBC nanocylinders may be lower than the actual height of the nanocylinders. Indeed, extinction spectra for a sample etched for even longer time showed close similarity to simulated spectra for even smaller TDBC cylinder heights (see Figure S8, Supporting Information for the measured spectra for the over‐etched sample and simulated spectra for different heights). The experimentally obtained broader peaks compared to the simulation can be attributed to the imperfect nanostructures, with slightly tilted sidewalls and less defined edges, as confirmed using SEM (Figure S3, Supporting Information) In addition, we cannot completely rule out possible weak signals from leftover TDBC nanopatterns between nanocylinders inside the array (Figure S9, Supporting Information). This may affect the extinction spectra especially when the signal from the nanocylinders themselves is comparably weak, thereby contributing to the discrepancy between the measured and simulated results for the smallest diameters.

**Figure 4:**
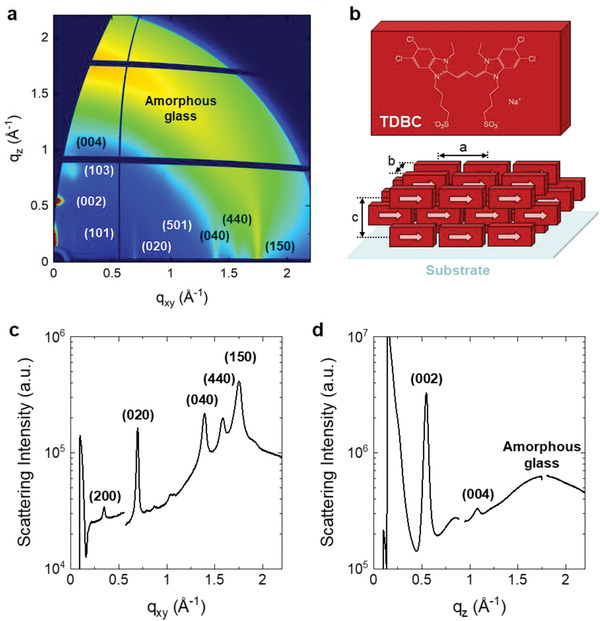
a) 2D GIWAXS image for a 200 nm thick TDBC film spin‐coated on a glass substrate. The incident angle was 0.16°, and the broad peak at *q* ≈ 1.8 Å^–1^ is from the amorphous glass substrate. b) 3D molecular orientations reconstructed from (a). 1D scattering profiles along c) in‐plane and d) out‐of‐plane directions.

We performed grazing incidence wide angle X‐ray scattering (GIWAXS) measurements to provide further details on the anisotropic nature of the TDBC films from which the nanocylinders are made (**Figure** **4**). Results for a 200 nm thick TDBC film on a glass substrate show clear anisotropic scattering peaks originating from the 3D J‐aggregate structure. From the GIWAXS peak analysis, the TDBC film could be successfully modelled as a body‐centered‐like structure as depicted in Figure 4b. The lattice parameters obtained are given by *a* = 35.6 Å, *b* = 18.0 Å, *c* = 23.3 Å. It is interesting to note that this configuration can maintain the characteristics of J‐aggregation, such as head‐to‐tail interaction and slip‐stacked arrangements with nearest neighboring molecules, in 3D.^[^ ^28^ ^]^ This indicates that TDBC molecules constitute crystalline domains with edge‐on orientation parallel to the substrate. In turn, their transition dipole moments are also aligned along the in‐plane direction (indicated by the arrows in Figure 4b), which inevitably leads to anisotropic optical properties as consistent with our ellipsometry results. The in‐plane (Figure 4c) and out‐of‐plane (Figure 4d) scattering data extracted from Figure 4a also support strongly anisotropic molecular orientation in the films with different lattice spacings.

**Figure 5:**
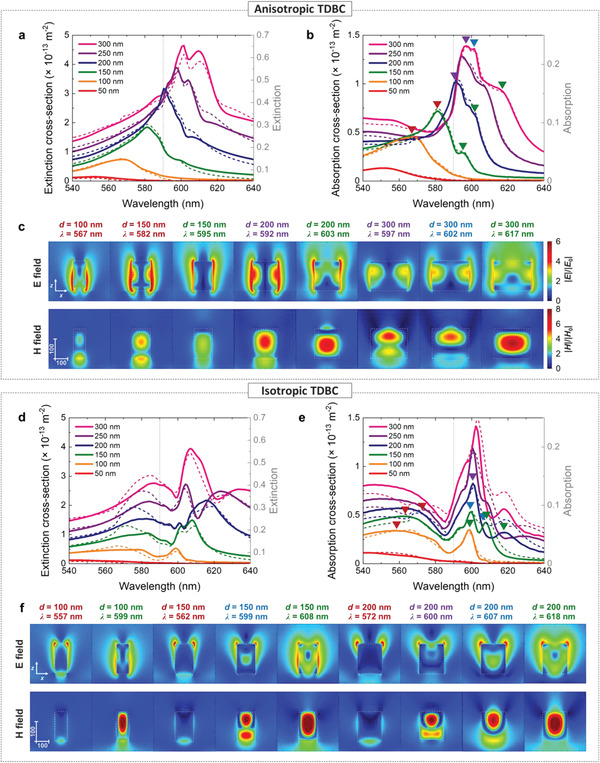
Calculated a) extinction and b) absorption cross‐section for single anisotropic TDBC nanocylinders. Solid curves indicate the extinction/absorption cross‐section for single nanocylinders and the dashed curves indicates the extinction/absorption spectra for array structures with periodicity of 800 nm. c) Representative electric (upper row panels) and magnetic (lower row panels) nearfield plots for *xz*‐plane. Axis directions and scale bars are in the leftmost panels. d–f) Results equivalent to (a–c) but for isotropic TDBC. The resonance positions are designated in (b) and (e) with the same color code.

To further investigate the characteristics of the nanooptical resonances in our system, we analyzed simulated electric and magnetic nearfields for each resonance. **Figure** **5**a,b shows extinction and absorption cross sections for single nanocylinders (solid lines), as almost identical to the extinction/absorption spectra for periodic arrays (dashed lines, periodicity of 800 nm as in our experiments). To clarify the effects of the anisotropic TDBC permittivity, we also present results for the same structures, but with fictitious isotropic permittivity (Figure 5d,e). Figure 5c,f illustrates the representative electric and magnetic nearfield profiles in the *xz*‐plane (i.e., cross sections through the middle of the nanocylinders at *y* = 0) of the TDBC nanocylinders for the wavelengths where the absorption cross‐section exhibits resonant peaks, as designated with colored inverted‐triangle markers in Figures 5b and 5e. For both cases, commonly noticeable resonances can be categorized using the color code as follows: 1) plasmon‐like LSEP resonances (designated with red markers and text), and 2) lowest order (green markers and text) and higher order Mie resonances (blue and purple markers and text). Henceforward, we discuss the nearfield profiles for the anisotropic TDBC nanocylinders (Figure 5c) and compare them with those for the corresponding isotropic TDBC nanocylinders (Figure 5f). First, nanocylinders with small diameters (*d* = 100 and 150 nm) seem to support LSEP resonances in the negative permittivity regions, showing dipolar nearfield profiles resembling LSPRs. However, in contrast to LSPRs of traditional (isotropic) metals, the electric field penetrates into the anisotropic nanostructure and induces a magnetic field near the center (two leftmost columns in Figure 5c). We particularly note a “cross‐hatch” pattern of the electric field in the anisotropic nanocylinders. This is indeed a unique feature for nanostructured Type II hyperbolic materials (*ε*_xy_ < 0, *ε*_z_ > 0), where the hyperbolic polariton propagation is directionally restricted at a fixed angle.^[^ ^29^ ^]^ Similar nearfield profiles have been reported in previous studies of hexagonal boron nitride (also natural hyperbolic material) nanocones.^[^ ^29^, ^30^ ^]^ The resonances in the negative permittivity region for the anisotropic TDBC nanostructures should therefore be referred to as hyperbolic LSEPs. On the contrary, the fictitious isotropic TDBC acts like a normal metal in the negative permittivity region and prohibits electric field penetration due to evanescent decay of the electromagnetic field into the nanostructure (columns with red texts in Figure 5f). As a result, the isotropic TDBC nanocylinders do not show the cross‐hatch pattern, but instead a remarkable resemblance with the nearfields of traditional gold nanocylinders (see Figure S10, Supporting Information). It is noteworthy that resonant peaks are observable in the negative permittivity region also for the larger isotropic nanocylinders (*d* = 200–300 nm) in contrast to the anisotropic nanocylinders, as attributed to different geometry‐dependence of LSEPs and hyperbolic LSEPs.

We now turn to the positive permittivity region of the TDBC, for which we observe clear Mie resonances for the larger nanocylinders. The lowest order Mie resonance has characteristic features of a magnetic dipole which consists of circulating currents and a consequent magnetic dipole moment along the *y*‐direction at the center of the nanocylinder (columns with green texts in Figure 5c,f). As the diameter increases, the magnetic dipole resonance redshifts due to the larger optical path length in the nanostructure. In case of isotropic TDBC, the Mie resonances become observable for smaller diameters. For instance, 100‐nm‐diameter isotropic TDBC nanocylinders can support the magnetic dipole resonance, whereas the same structure made of anisotropic TDBC cannot. We attribute this effect to the anisotropic TDBC providing effectively shorter optical path length along the *z*‐direction in the large positive permittivity region (*ε*_z_ < *ε*_xy_, see Figure 3b), such that larger dimensions are necessary to support the same type of resonances for the anisotropic TDBC compared to isotropic TDBC. This consistently applies also to the higher order Mie resonances, which are also clearly visible in both the anisotropic and isotropic TDBC nanocylinders (columns with blue and purple text in Figure 5c,f). We take the electric dipole as an example, which corresponds to the second lowest order Mie resonance with concentrated electric dipole moment along the *x*‐direction at the center and magnetic multipoles (columns with blue text in Figure 5f). While this resonance appears for the isotropic TDBC nanocylinders from diameters of 150 nm and larger, this characteristic feature is not clearly visible even for the largest anisotropic TDBC nanocylinder. In general, the resonances in the positive permittivity region for the anisotropic TDBC nanostructures may be referred to as elliptic Mie resonances. It is worth noting that the Mie resonances for the isotropic TDBC nanocylinders show almost identical nearfield profiles to those for silicon nanocylinders with the same dimensions (see Figure S11, Supporting Information).

We also note that the peaks might seem to smoothly shift across the exciton resonance with increasing diameter. However, the hyperbolic LSEP resonances and elliptic Mie resonances cannot cross the exciton resonance (vertical dashed line) where the permittivity changes sign (i.e., epsilon near pole). Thereby, this finding should rather be understood as the disappearance of one type of resonance and the appearance of another when crossing the exciton resonance position. In this respect, the nearfield cross‐hatch pattern forms a signature of hyperbolic polaritons and becomes a key feature to identify the type of resonances. We note that the nearfield profiles for both resonances in the negative permittivity region (marked in red) show these cross‐hatch patterns whereas those in the positive permittivity region (marked in green, blue and purple) do not (see Figure 5c). Interestingly, the nanocylinders with diameter of 150 nm simultaneously support both resonances as shown in Figure 5a,b, providing dual functionality in a single nanostructure. For situations where isotropic properties are desired, it may be possible to suppress the anisotropy by dispersing the TDBC in a PVA matrix at low concentrations. Indeed, such TDBC systems have been explored for other applications^[^ ^31^, ^32^, ^33^ ^]^ and this can be one reason why previous studies did not report the possibility to obtain anisotropic TDBC and corresponding permittivity. Additional stability tests on the excitonic nanostructures studied in this work showed robustness against the light illumination and the storage at ambient conditions (Figures S12 and S13, Supporting Information).

## 3 Conclusions

In summary, we report the experimental demonstration of TDBC nanostructures and their nanooptical properties, realized using a novel fabrication approach based on SANE assisted by RIE. Interestingly, extinction measurements of resulting cylindrical nanostructure arrays showed clear optical resonances not only in the spectral region with negative in‐plane permittivity, but also on the other side of the exciton resonance where the permittivity is positive. We further found the TDBC thin films to be highly anisotropic, leading to hyperbolic properties in the region with (in‐plane) negative permittivity and elliptic properties in the positive permittivity region. The organic excitonic nanostructures could therefore support hyperbolic LSEP resonances and elliptic Mie resonances in these two different spectral regions, respectively. Neither of these types of resonances were previously reported for this excitonic material, which has typically been treated as isotropic. Optical simulations based on measured anisotropic TDBC permittivity successfully reproduced the experimental results, and gave further insight on the optical nearfields. Our study highlights organic excitonic materials as promising building blocks in nanooptics and adds anisotropic TDBC to the palette of hyperbolic organic materials.^[^ ^8^, ^34^, ^35^ ^]^ Combined with the presented nanostructuring method, our study takes important steps towards functional metasurfaces and other nanooptical applications based on organic excitonic nanoantennas. Not least, the dual functionality and anisotropic optical properties in excitonic nanoantennas may find uses in various applications ranging from wavelength‐selective flat optics or biosensors to natural hyperbolic metasurfaces operating at visible frequencies.^[^ ^36^, ^37^ ^]^ The fabrication method may also open for nanostructuring and nanooptical studies of a variety of other organic materials, including other excitonic materials as well as conducting polymers.^[^ ^8^, ^38^, ^39^ ^]^

## Conflict of Interest

The authors declare no conflict of interest.

## Supporting information

Supporting Information

## Data Availability

The data that support the findings of this study are available from the corresponding author upon reasonable request.
