# Supplementary material for: Organic Anisotropic Excitonic Optical Nanoantennas
Source: Adv Sci (Weinh). 2022 May 26;9(23):2201907. doi: 10.1002/advs.202201907 (PMC9376850; doi:10.1002/advs.202201907)
Supplement: Supplementary file 1 — Supporting Information [file ADVS-9-2201907-s001.pdf]

Supporting Information

Organic Anisotropic Excitonic Optical Nanoantennas

Evan S. H. Kang,[1,2] Sriram KK,[3] Inho Jeon,[1] Jehan Kim,[4] Shangzhi Chen,[2] Kyoung-Ho Kim,[1] Ka-Hyun Kim,[1] Hyun Seok Lee,[1] Fredrik Westerlund,[3] and Magnus P. Jonsson[2]**

[1]Department of Physics, Chungbuk National University, Cheongju 28644, Republic of Korea

[2]Laboratory of Organic Electronics, Department of Science and Technology (ITN), Linköping University, Norrköping 60174, Sweden

[3]Department of Biology and Biological Engineering, Chalmers University of Technology, Gothenburg 41296, Sweden

[4]Pohang Accelerator Laboratory, Pohang University of Science and Technology, Pohang 37673, Republic of Korea

*eshkang@chungbuk.ac.kr, *magnus.jonsson@liu.se

Materials and methods

Fabrication of Si master templates

Si master templates with the inverse structure of the final nanocylinder arrays were prepared using e-beam lithography. To start with, a 2 μm thermal oxide layer was grown on a fresh 4" Si wafer of 500 μm thickness (N-type, Si-Mat) using an oxidation furnace (Centrotherm, 1050°C, 780 minutes). Alignment marks to assist electron beam lithography of nanostructures were first obtained through photolithography (PL, S1813, Shipley Inc., USA) and reactive ion etching (RIE, 50 sccm Ar, 50 sccm CHF_3, 150 W RF-power, 30 mbar pressure) to obtain ~900 nm deep features, verified using a surface profiler (Dektak D150, Veeco instruments). After RIE, the

substrate was cleaned using piranha solution (con. H_2SO_4 and H_2O_2 at 2:1 ratio, 120°C for 10 minutes), rinsed with deionized water and dried using a nitrogen gun. Subsequently, a 20 nm thick chromium layer was deposited using e-beam evaporation (AVAC HVC600), followed by sputtering of 24 nm thick SiO_2 (FHR MS-150 Sputter) to form a hard mask for RIE of nanostructures. Electron beam lithography (EBL, JEOL JBX-9300FS) was carried out using AR-P 6200.13 resist (diluted in Anisole, 1:1 ratio, Allresist GmBH) and then etched using RIE (50 sccm NF_3, 25 W RF-power, 8 mbar pressure) to get nanostructures of desired depths. Sputtered SiO_2 was removed with a CF_4 etch (Plasmatherm RIE, 40 sccm CF_4, 100 W RF-power, 100 mT pressure) and chromium layer was removed using a chromium etchant (SunChem AB, Sweden). The substrate was again cleaned using piranha solution as described above, to obtain the master structure shown in Figure S1.

Fabrication of TDBC nanostructures

Polyurethane acrylate (PUA) molds with the same structure were fabricated by two rounds of replication of Si master. 5,6-Dichloro-2-[[5,6-dichloro-1-ethyl-3-(4-sulfobutyl)-benzimidazol-2-ylidene]-propenyl]1-ethyl-3-(4-sulfobutyl)-benzimidazolium hydroxide, inner salt, sodium salt (TDBC, Few Chemicals), was used to make a solution of 30 mg/ml dissolved in DI water. The TDBC films were prepared by spin-coating the solution at 2000 rpm for 90 s onto a pre-cleaned glass substrate (sonicated in cleaning detergent, deionized water, acetone and isopropanol for 10 min respectively, followed by oxygen plasma treatment at 200 W for 5 min). Next, photoresist (AZ5412E, diluted with propylene glycol monomethyl ether acetate by 1:5 volume ratio) was cast on the PUA mold, and then directly sandwiched between the mold and the TDBC film. After drying at 50 °C (sufficiently low temperature not to affect the exciton resonance) for 30 min and

subsequent cooling down to room temperature, the detachment of the mold left the photoresist nanopatterns on the TDBC film. Reactive oxygen plasma etching (12 sccm, 50 W) for 1-2 min was applied using the photoresist nanopatterns as a mask. Depending on the thickness of the original TDBC film, the etching time can be varied to completely remove the TDBC interspaced between nanocylinders.

Optical measurements

The extinction spectra for TDBC nanocylinder arrays were measured using a microscope (BX51, Olympus) coupled to a VIS-NIR spectrometer (Ocean HDX-VIS-NIR, Ocean Insight) via an optical fiber. The fiber entrance acting like a pinhole limits the detected signal to the area with a diameter of approximately 16 μm in the sample. This is not only sufficiently large to contain several hundreds of nanocylinders, but also sufficiently small compared to the size of the square array of nanocylinders (a side length of 125 μm), enabling the measurement of the signal only from the nanocylinders inside a single array. A tungsten halogen lamp and a linear polarizer installed in the microscope were used as a linearly polarized light source. Transmission (T) was measured to determine the extinction ($E = 1 - T$).

Ellipsometry

TDBC films were deposited on 2-inch single-side polished sapphire wafers (0.43 mm thickness, Semiconductor Wafer Inc., Taiwan). Ellipsometric data (Ψ and Δ) were obtained by a variable angle spectroscopic ellipsometer of the dual rotating compensator type (RC2, J. A. Woollam Co., Inc.). All measurements were performed in air at room temperature for five different incident angles (50°, 55°, 60°, 65°, and 70°) and in the wavelength range of 210 nm – 1000 nm in steps of

1 nm, and 1000 nm – 1690 nm in steps of 2.5 nm. The data were analyzed by WVASE32® software (J. A. Woollam Co., Inc.). Using the thickness of TDBC directly obtained by the surface profiler and AFM, a point-by-point fitting method was employed to determine the optical constants, where the real and imaginary permittivity values were fitted separately at each wavelength. In the analyses, the model parameters were varied to obtain the best fit between experimental and model-generated data by minimizing the mean-square error.

Finite-Difference Time-Domain (FDTD) Simulations

FDTD Solution (Lumerical) was used for the simulated spectra and nearfield plots. The anisotropic complex permittivity of TDBC was determined by ellipsometry and imported for the simulations. The simulation consists of an optically thick glass substrate (refractive index of 1.5) and a TDBC nanocylinder on top. A plane-wave polarized in x axis with a wavelength range of 400-800 nm was used as a source. The mesh size was set to 5 nm for all three axes. For periodic nanocylinder arrays, the simulation size was 800 nm in x, y, and z axis. Anti-symmetric, symmetric and perfectly matched layers (PML) boundary conditions were respectively used for x, y and z axis to establish the periodic nanocylinder array in the xy-plane. Transmission (T) and Reflection (R) monitors were installed on both sides of the nanocylinder to calculate the extinction ($E = 1 - T$) and absorption ($A = 1 - R - T$). For single nanocylinders, the extinction and absorption cross-sections of isolated TDBC nanocylinders were obtained using total-field scattered-field. The electric and magnetic nearfield plots were obtained using frequency domain field profile monitors.

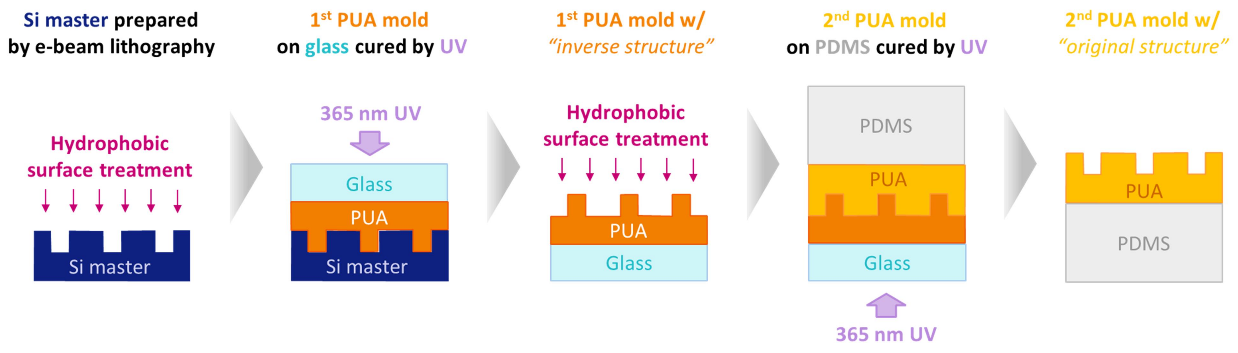

Figure S1. Fabrication scheme of PUA stamps from the silicon master structure. Double replication resulted in the same structure as the original structure.

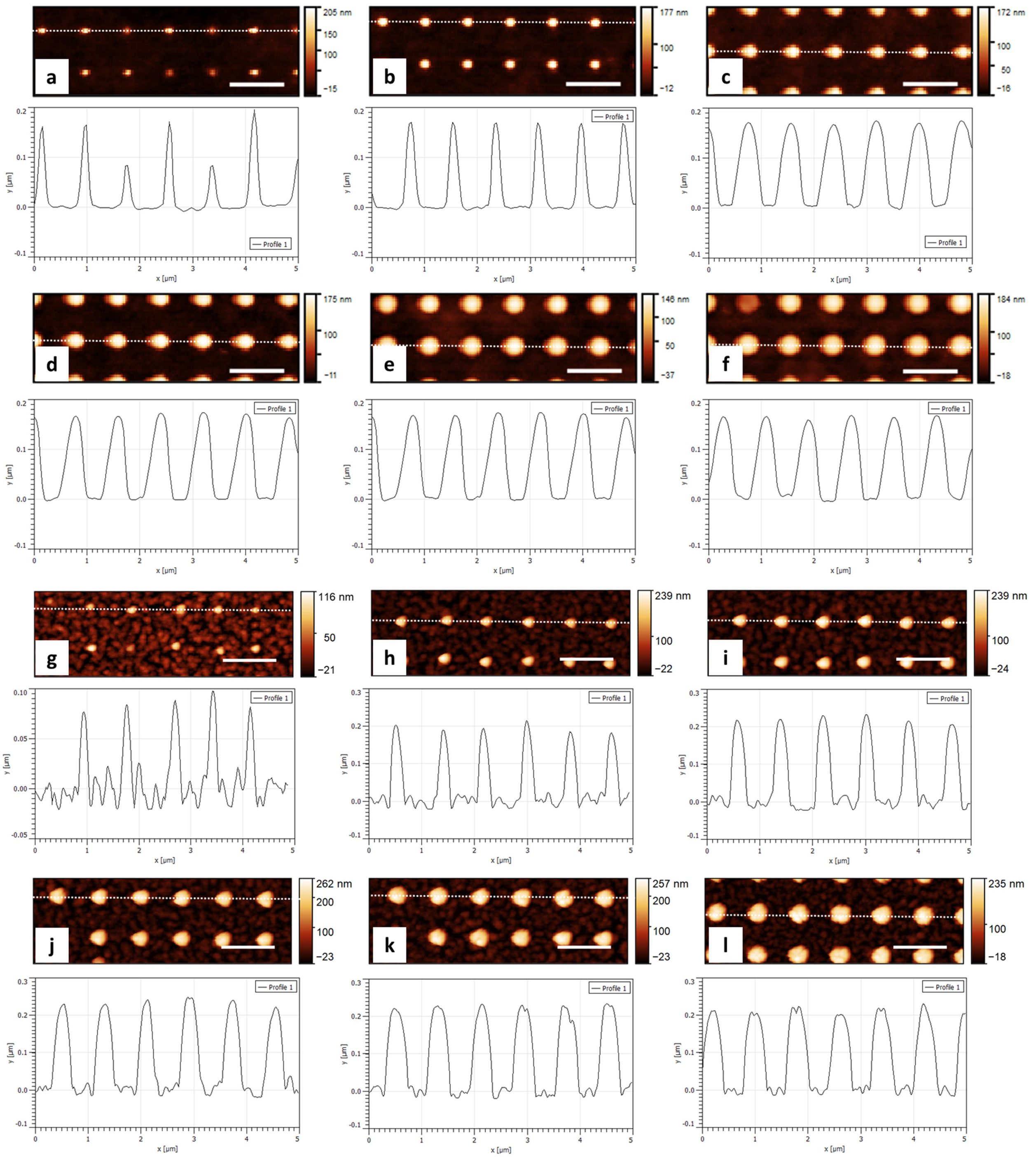

Figure S2. AFM images and height profiles along the dashed lines (a-f) for PR nanopatterns fabricated using SANE on TDBC and (g-l) for the resultant TDBC nanocylinder arrays after RIE. The diameters were 50, 100, 150, 200, 250, 300 nm, respectively. The scale bars are all 1 μm.

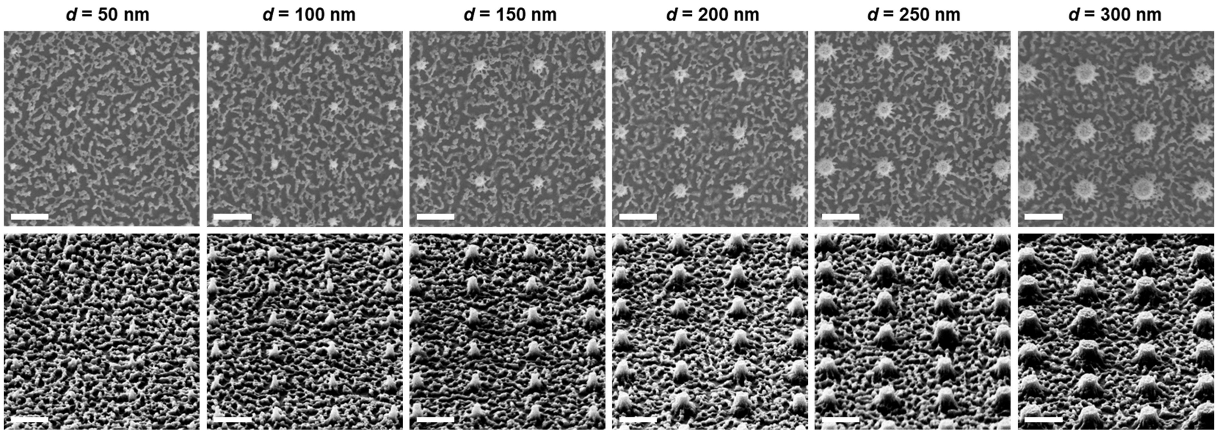

Figure S3. Top view (upper panels) and 67° tilted view (lower panels) SEM images of the TDBC nanocylinder arrays. The scale bars are all 500 nm. 2 nm thick Pt was sputtered on top to reduce the charging effect.

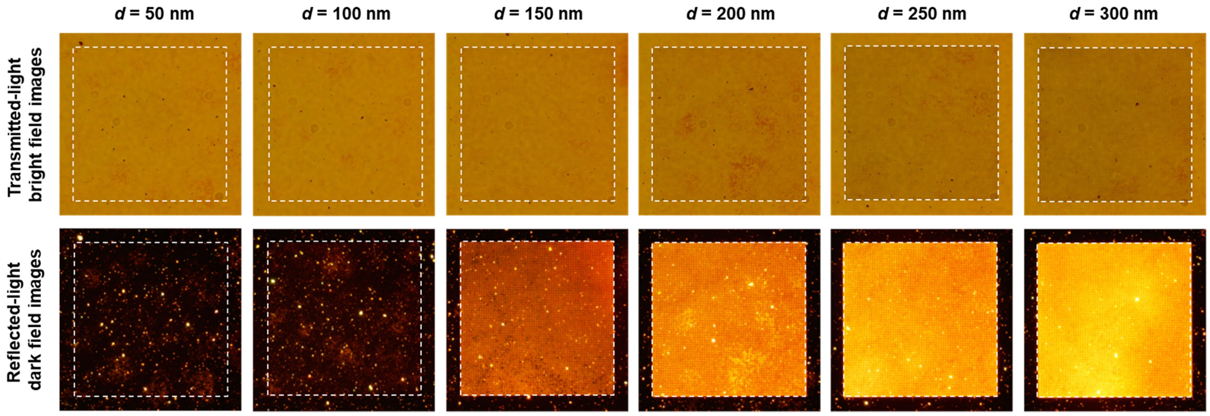

Figure S4. (a) Transmitted-light bright field and (b) reflected-light dark field optical microscope images for TDBC nanocylinder arrays. Array areas are designated by white dashed boxes. The lengths of one side of arrays are 125 μm.

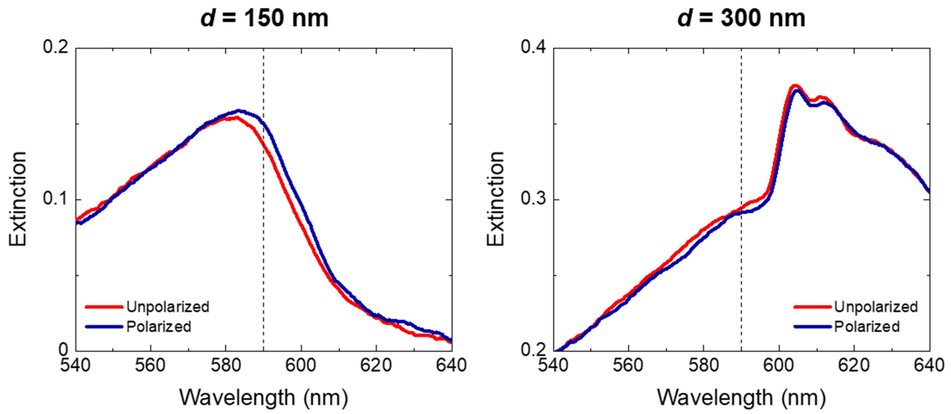

Figure S5. Extinction spectra for (a) hyperbolic polariton resonance for d = 150 nm and (b) elliptic Mie resonances for d = 300 nm using unpolarized and polarized incident light. The polarization direction was along the array axes.

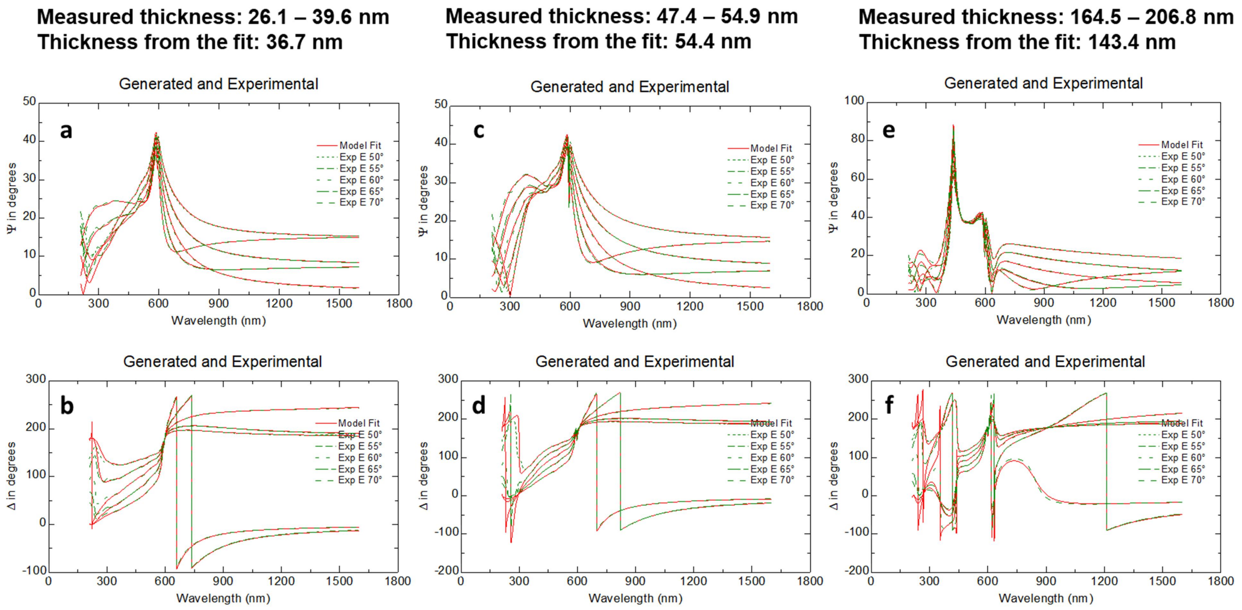

Figure S6. Ellipsometric raw data ψ and Δ of TDBC thin films with three different thicknesses.

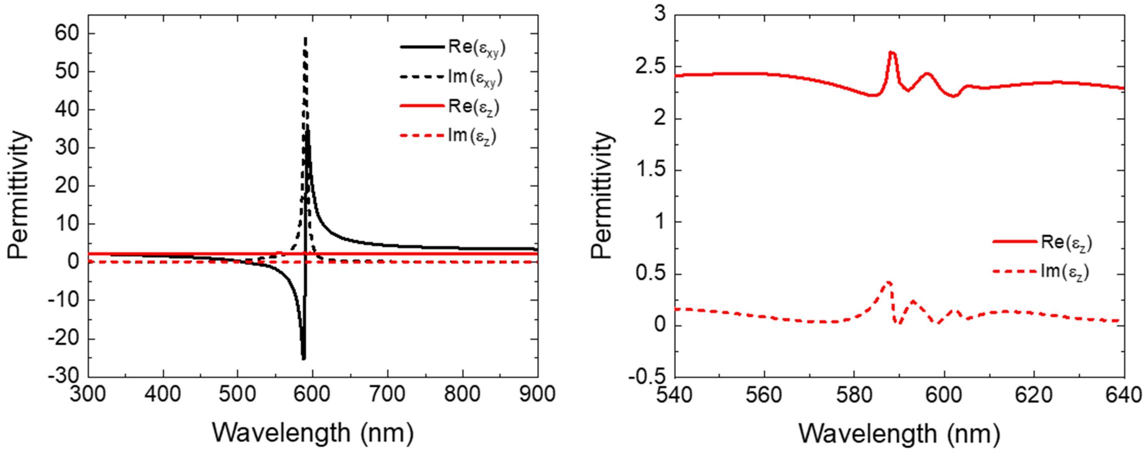

Figure S7. Complex permittivity of TDBC as obtained from ellipsometry measurements. The right panel shows an enlarged out-of-plane permittivity (ε_z) for clarity.

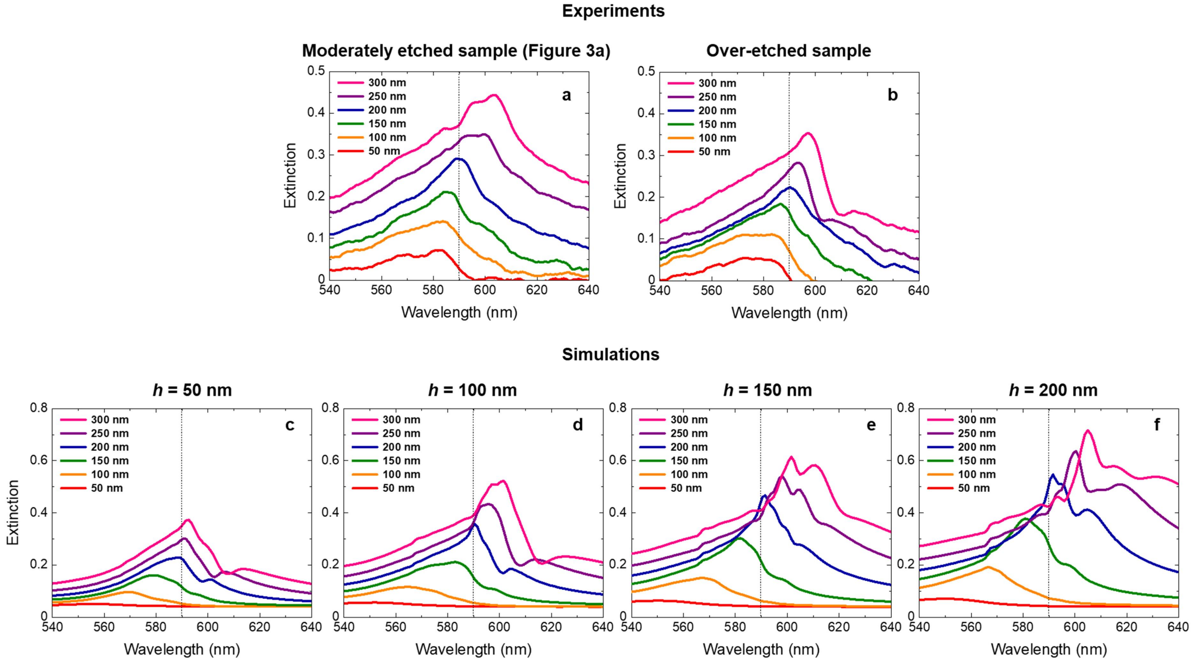

Figure S8. Measured extinction spectra for TDBC nanocylinder for (a) a moderately etched sample (same as Figure 3a) and (b) an over-etched sample. (c-f) Calculated extinction spectra for anisotropic TDBC nanocylinder arrays of various heights h = 50-200 nm.

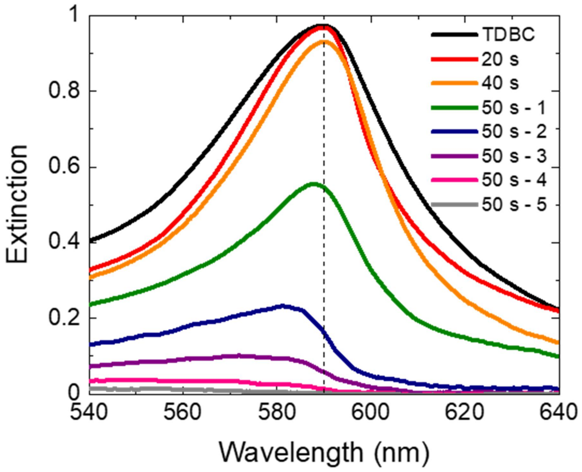

Figure S9. Extinction peak change after successive RIE on a 30 nm thick TDBC film with an 80 nm thick PR film on top. The etched sample resembles the area between nanocylinders. The legend indicates cumulative etching times. The off-exciton extinction peaks may be due to the background leftover nanopatterns, as observed in AFM (Figure 2) and SEM images (Figure S3). The five curves for 50 s (from green to gray) were obtained from different areas of the same sample, showing a possibility of nonuniform etching in large areas.

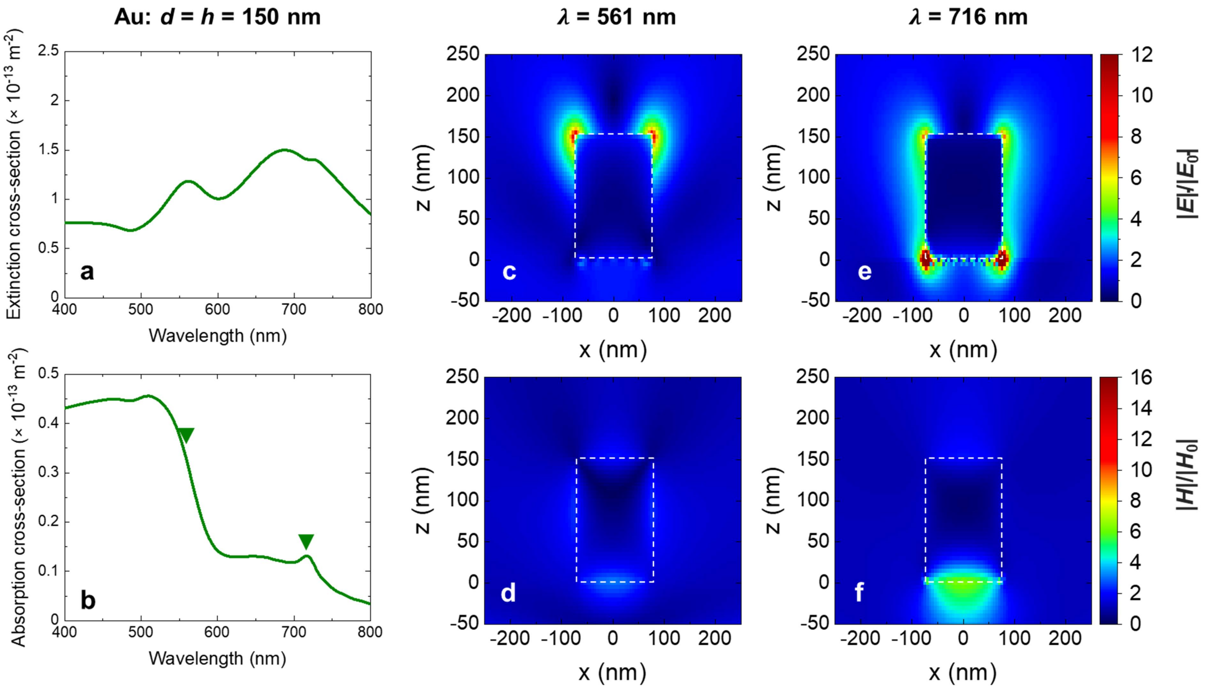

Figure S10. Simulated (a) extinction cross section and (b) absorption cross section for a single gold nanocylinder with $d = h = 150$ nm. (c-f) Electric (upper panels) and magnetic (lower panels) nearfields at the wavelengths designated in (b).

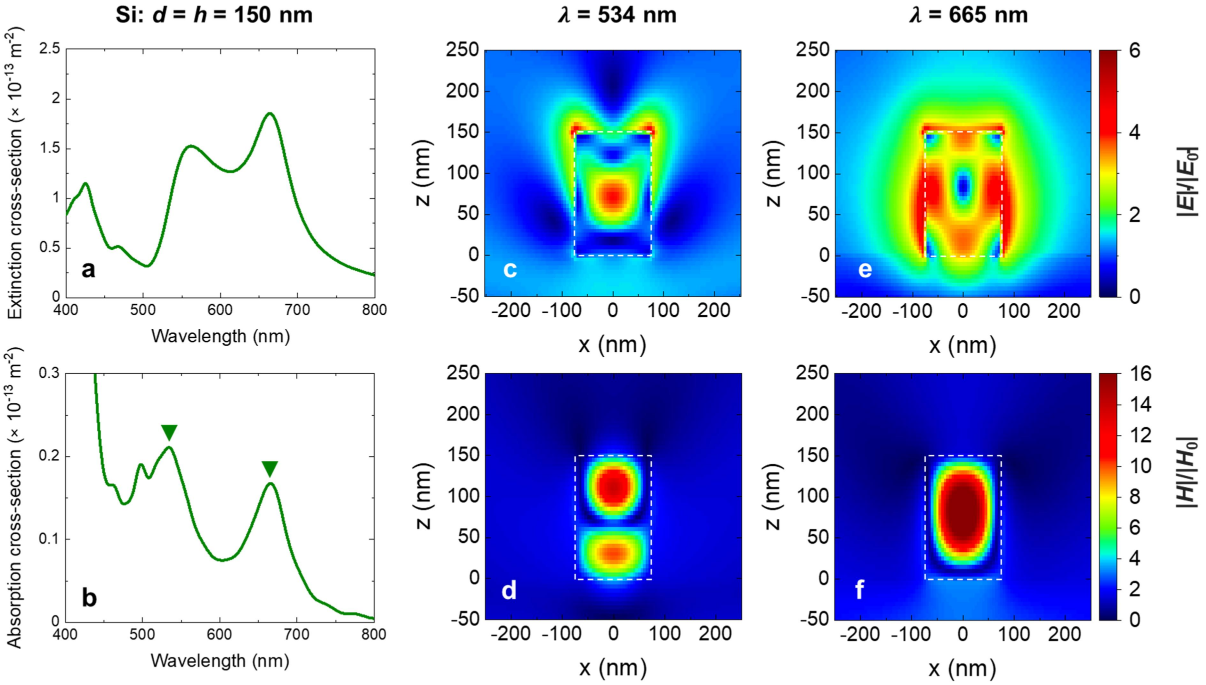

Figure S11. Simulated (a) extinction cross section and (b) absorption cross section for a single silicon nanocylinder with d = h = 150 nm. (c-f) Electric (upper panels) and magnetic (lower panels) nearfields at the wavelengths of the absorption peaks designated in (b).

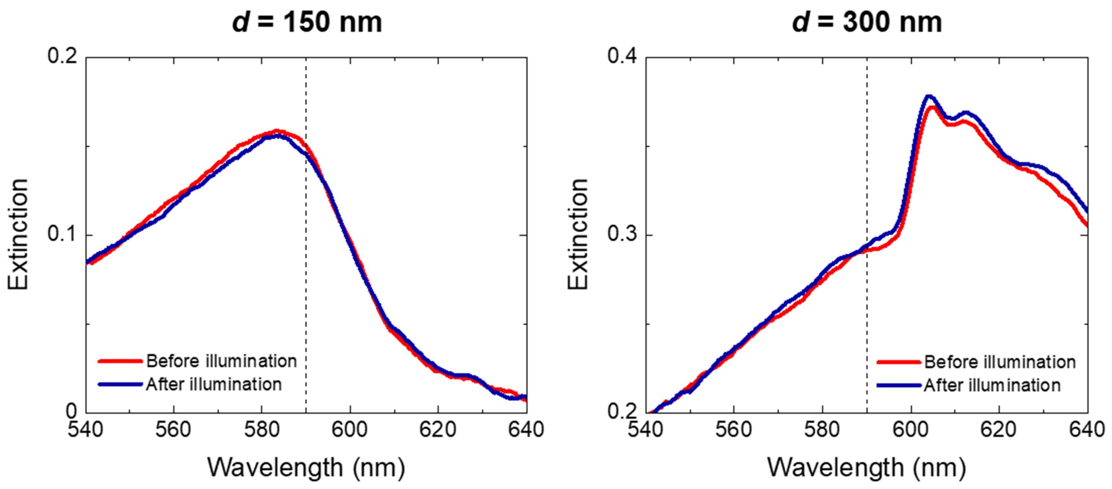

Figure S12. Extinction spectra for (a) hyperbolic polariton resonance for d = 150 nm and (b) elliptic Mie resonances for d = 300 nm before and after 1 hour of light illumination by the halogen lamp (~24 mW/cm^2 at 590 nm).

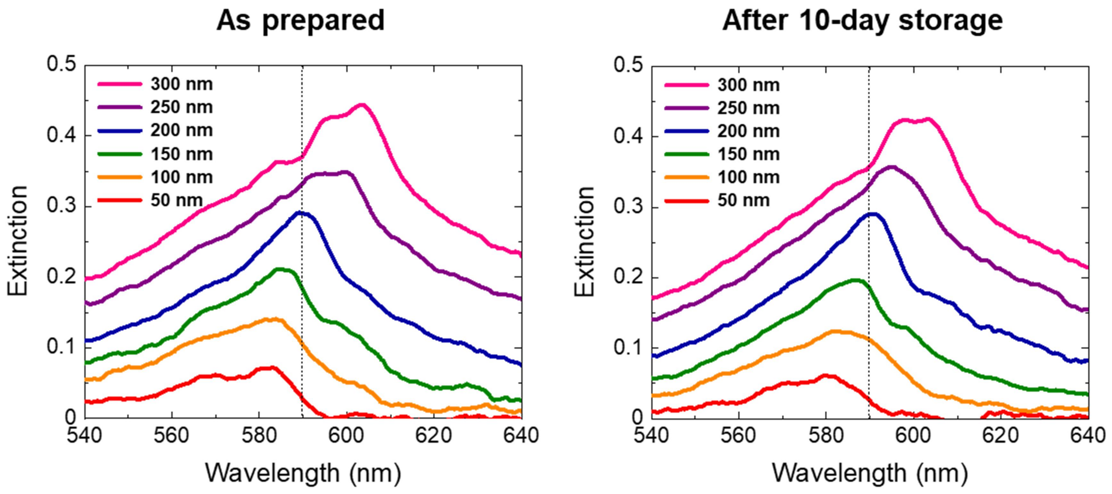

Figure S13. Extinction spectra for TDBC nanocylinders (a) as prepared and (b) after 10 days of storage under the ambient condition.
